# Characterization of anti-malarial resistance genes in pregnant and non-pregnant subjects of the northwest of Colombia

**DOI:** 10.1186/1475-2875-13-S1-P100

**Published:** 2014-09-22

**Authors:** Eliana Arango, Jaime Carmona, Amanda Maestre

**Affiliations:** 1 Grupo Salud y Comunidad, Facultad de Medicina, Universidad de Antioquia, Medellin, Colombia

## Background

Surveillance of resistance to antimalarials is a priority in the light of limited therapeutic options against *P. falciparum* and the emergence of artemisinin resistant strains. In some countries, antifolates and 4-aminoquinolines might remain a second line choice therapy, mainly in particular clinical situations or when availability of artemisinin combined therapy (ACT) is limited. During the past decade, we have monitored the status of antimalarial resistance associated genes in the northwest region of Colombia, where both vivax and falciparum malaria are highly endemic. Treatment for *P. falciparum* infection in Colombia is based on ACT, and for *P. vivax*, on choloroquine plus primaquine. Some authors have reported pregnant subjects as an important reservoir of *P. falciparum* resistant clones, mainly as a consequence of Intermittent preventive treatment in pregnancy (IPTp). In the light of the recent reports made in Colombia of a higher than expected frequency of pregnancy associated malaria, efforts were directed to study the genetic characteristics of the *P. falciparum* and *P. vivax* alleles associated with antifolate resistance in pregnant and non-pregnant populations of the country.

## Materials and methods

A sample size sample of 20 isolates of *P. vivax* and 20 of *P. falciparum* was proposed and grouped into 1) parturient with a positive peripheral blood test, 2) positive placental blood, 3) gestational malaria (non-parturient) and 4) positive non-pregnant subject. The genes *dhfr*, *dhps* and *mdr1* were assessed for both species. For this, PCR followed by direct sequencing was performed in each gene. Analysis of the sequences was carried out using Genious 6.1.3.

## Results

Successful DNA amplification was observed in 20 *P. vivax* and in 15 *P. falciparum* samples. In 19/20 *P. vivax*, the triple mutant *Pvdhfr* (58R, 117N) and *Pvdhps* (383G) was detected. Similarly, for *P. falciparum*, 14/15 isolates exhibited the triple mutant genotype *Pfdhfr* (51I, 108N) and *Pfdhps* (437G). Assessment of the *Pvmdr1* gene confirmed presence of the wild type alleles Y976 and F1076 in 19/20 samples. A double mutant (976F, 1076L) was observed in a pregnant woman. As for *Pfmdr1*, all samples presented the haplotype N86, 184F. Distribution of the different genotypes was similar in all groups.

## Conclusions

Over 90% of *P. falciparum* samples, regardless of the group of study, exhibited mutant alleles of *Pfdhps*, *Pfdhfr* y *Pfmdr1*. *Pvmdr1* remains wild-type in all groups studied. A triple-mutant pattern of resistance in antifolat-related genes was observed in *P. vivax* and *P. falciparum*. This renders antifolates a non-viable option for a IPTp policy in the country.

